# Report on Caries of the Teeth

**Published:** 1850-01

**Authors:** B. T. Whitney

**Affiliations:** Clarksville, Tenn.


### DENTAL REGISTER OF THE WEST.

VOL. III. JANUARY, 1850. NO. 2.

### REPORT ON CARIES OF THE TEETH,

AND ITS TREATMENT; SUBMITTED TO THE MISSISSIPPI VALLEY ASSOCIATION OF DENTAL SURGEONS, AT THEIR FIFTH ANNUAL MEETING, HELD IN LOUISVILLE, KY., 2d Tuesday of Sept., 1849:

BY DR. B. T. WHITNEY.

There is no disease, which the dental apparatus is heir to, of a more disastrous character than caries of the teeth; and none more common, and at the same time, more under the control of the dentist, by timely and proper treatment. It is so common, that we have only to look into the mouth of almost every person over seven years of age to find the disease in question; and so disastrous, that but few, who have lived more than sixteen years without the aid of a dentist, but that can bear witness of its ravages.

A disease so general and destructive to organs so important, requires the most careful and attentive investigation of every practitioner of the dental art. It has been estimated by persons, who have paid some attention to the subject, but with how much truth, it would not become me to say, that more teeth were lost than saved, by "*professional*" interference. If this be true, it is a sad commentary on the worth of the profession; or shows that the greater amount of business is in the hands of the empiric.

The teeth of a person perfectly healthy in childhood, fully developed, and well constituted, are subject to decay, only from external agencies, acting directly on the substance of the tooth. Changes in health after adult age, even the frequent attacks of disease, or fevers of a low type, do not materially effect the teeth. Their texture is permanently fixed with the child, and not vacillating in after life; with the health of the individual. They may become the seat of disease, but not constitutionally; but from local causes or secondary results. But if, in departing from natures course, and transgressing her laws, these organs become constitutionally defective or diseased, the hope of restoring them to health, or bringing about what nature failed to do in its organization, is vain; but if only secondarily, or from local causes, they take on disease, we have only to direct our treatment to the removal of the causes producing it, and repair the injury already sustained; and we accomplish the desideratum.

In many cases, the seeds of disease, and imperfect development of these organs are sown in childhood. Attacks of disease, enfeebled state of the health, or improper diet during ossification, may cause imperfect development of the teeth, frail texture, or atrophy; and neglected dentition, may produce malposition or disease of the teeth; and thin or imperfect enamel. Decay and premature loss of the teeth, must be the result in either case, without the greatest care.

The power of the teeth to resist causes of decay, is in proportion to their organization—whether hard or soft; and position, whether regular or irregular; and require preventive or curative means, or they fall victims to caries, in the same ratio. This holds good with teeth in the same mouth, as well as with different persons; as the supply of bony matter may be stopped or impaired during the formation of some teeth, and abundant at other times. This is evident from teeth decaying in pairs; the remote causes, acting alike on those formed at another time, without injury.

To successfully apply the preventive means, requires an accurate knowledge of the causes producing caries of the teeth; and though numerous, each deserve a separate chapter.

There are often remote causes acting upon the teeth; but more generally they are palpable. In well formed and regular dentures, of good physical condition, the want of cleanliness, is, perhaps, the most general; next to this, abuses and accidents, by which the enamel becomes fractured or abraded, so as to expose the dentine to the corroding effect of all foreign agencies, so often brought in contact with the teeth, or morbid action of the system. Many articles of medicine may act chemically on the teeth. The free and careless use of acids, will speedily neutralize the lime of the teeth, and hasten their destruction. The use, even of a tube to convey it to the throat does not entirely protect them; as in swallowing, it regurgitates against the teeth. The use of improper toothpicks and dentifrices are often as injurious to the teeth as the lack of cleanliness. The injudicious use of the file, scraping the teeth, improper adaptation of artificial substitutes, and careless plugging or of bad material, or the more common mal-practices of the *tooth destroyer*, alias, dentist; directly producing, what the unfortunate had employed them, in good faith, to cure.

We have many things to take into consideration when called upon to treat caries of the teeth, besides plugging up the cavities. Some diseased action of the system, or mercurial influence, may have rendered the fluids of the mouth impure. That action of the system may have passed off; but the unhealthy condition of the mouth, has, in turn, more permanently vitiated the saliva; and its effects are visible on the teeth. We may find chronic inflammation of the gums, calcarious deposit, ulceration of the peridental membrane or pulp, absorption of the alveolar process, and loosened and dead teeth—really a motly assemblage of disease presenting itself to our view.

But before proceeding to perform that very important operation, we must perform the more important one, of restoring health to the mouth; otherwise, our efforts to save the teeth will prove but temporary, or entirely fail. To designate and carry out the proper treatment for such a complication, requires knowledge, experience, and skill. It is often a sad report to the timid, when only expecting to have a few teeth plugged, to be informed, that before performing that operation, it becomes necessary to extract dead teeth or roots, remove tartar, cure sponginess of the gums, allay irritation, and correct the morbid action of the salivary glands; thereby, subjecting him to a painful operation, and days or weeks treatment. Professional capacity, and gentlemanly deportment will, at once, inspire confidence; and by kindly controling the caprices of the patient, we soon gain the mastery, and are left to the free exercise of our own judgment and skill, in the use of curative and preventive means; and when we dismiss the patient, it is with confidence that the greatest good has been accomplished; and with gratification and self-respect, we can say with Cæsar, *veni, vidi, vici.*

In passing judgment on carious teeth, the professional knowledge of the operator is called out, to determine which teeth may be saved by plugging; as well as his firmness of character, respect for himself, and regard for his patient. He may be led to act in mercy to the imploring patients, against his better judgment; and after weeks, or even months, will have the mortification of being compelled, not only of removing the plug for the seventh time, but finally the offending tooth. It should be put down as a general rule, that in treating complicated caries, especially of the molar teeth, that it is much easier controling them on our table, than in the mouth; and should proceed at once to extract them. There may be an occasional exception. The incisors, cuspidati, and even bicuspidati, if the soft parts are healthy, the walls of the cavity good, and not too great a loss of substance, may be saved by judicious treatment.

The tooth is destroyed by the exposure of the osseous tissue, to the influence of external agencies; and so long as the juices of the mouth or liquids taken into it, and allowed to remain in contact, the decay must progress. In a healthy state, the enamel is protection, and so long as it is perfect, we have nothing to fear; but if the natural covering becomes destroyed, we are called upon to build up an artificial one; and when the operation is well performed, we have confidence in its permanency. To accomplish this, the cavity must be well cleaned of all carious portions of tooth, and of proper shape, however tedious and perplexing the operation may be; presenting a firm and healthy appearance, the foil well packed, perfectly solid, and finely polished, with the edges of the plug in firm contact, and even with the surface of the wall of the cavity; so as to present a smooth and uniform appearance, and prevent the admission of moisture, or the lodgment of foreign matter, or the mucous secretions of the mouth.

The treatment for caries of the teeth, is divided into two classes—filing and plugging. To arrest superficial caries of the lateral surfaces, the file alone may be sufficient; and it also is necessary in preparing the way for plugging.

The performance of the operation of filing, demands judgment as well as dexterity and skill; and when that instrument is properly used, it is one of the most important curative means in manual dentistry, and at the same time, if abused, is productive of as great evil as any instrument can be, in the hands of the empiric. If it is for the cure of superficial caries, it must be carried to the full extent necessary for the removal of all the discolored portions of the tooth; and at the same time in such manner as to preserve the beauty and symmetry of the organ; or if preparatory to plugging, by making space to enable the operator to get at the diseased portion of the tooth, or for the purpose of cutting down the ragged and weakened walls of the cavity; it should be used liberally, as the facility in performing, and the success of the other parts of the operation, mainly depends on this. The patience of the operator is often as much taxed as his skill, in controlling the caprices, or allaying the fears of the subject. In filing the incisors, if both teeth are decayed, we use a thin separating file, cut on both sides and edges, carrying it firmly and steadily between the teeth to near the gum, leaving, at that point, a slight projection or shoulder on each tooth, to prevent them approximating each other again. A thin, safe sided file, (one side smooth, so as to save the edge of the neighboring tooth,) is then carried diagonally, from right to left, or vice versa, cutting away more in the palatine than labial side of the tooth. In this manner, as much may be filed away as desired, without materially injuring, but sometimes improving the appearance of the front of the tooth, and affording sufficient room for the easy excavation of the cavity, and introduction of the foil. Where the file is used, the surface should be smoothed off with a half worn file or pummice stone, so that foreign matter, or the secretions of the mouth may not lodge there. If but one tooth is decayed, the safe sided file only should be used, that the sound tooth may be unharmed; otherwise the operation should be performed in the same manner. If it is on the approximal side of the bicuspidati or molares, the space made should much resemble the letter V, though not brought to a point at the gum. For this purpose, a file much thicker at one edge than the other, or like a clockmaker's pinion file, is necessary. It is important that we obtain this shape by filing, for two reasons: The decay, if much advanced, undermines the corners, or grinding surface of the tooth, which is liable to be broken off in mastication; and leaves firm and healthy walls; and secondly, it enables the operator to thoroughly clean and prepare the cavity, and insert the plug in the best possible manner. A third may be mentioned for the especial benefit of those, who think more of an economical operation than permanent good to the patient; and by carrying out the proper practice in such cases, less gold is required, and time and labor saved in filling the cavity. After the foil is well packed, the use of the file becomes necessary again, in cutting down the projecting portions of the plug, leaving a uniform surface, preparatory to the last part of the operation, that of finally polishing the plug.

The peculiar kinds and shape of files, or numerous instruments for excavating and preparing the cavity for the plug, need not be mentioned in this paper, as they are found in all varieties with the manufacturers or venders: and the operator should be supplied with a sufficient number of various patterns and sizes, together with drills and burrs, so as never to be at a loss or embarrassment in preparing any cavity. But for the easy and speedy accomplishment of this operation, a peculiar tact and much experience is necessary. It is often tedious and perplexing, from the peculiar location or condition of the disease. There can be no specific or definite rules laid down, by which the operator may proceed in using the instruments, or finally accomplishing the desirable object for all the peculiar cases; and can only be accomplished by study, ability and perseverance. Four things at least, should be strictly observed in this part of the treatment of caries; the discolored portion should be entirely removed, the walls of the cavity firm and healthy, with smooth and uniform edges, the inner part of the cavity at least as large as the orifice, and if shallow, with some opposing depressions, to aid in retaining the plug; and finally thoroughly cleansing and drying the cavity. However great the labor or time employed, we should never stop short of fully accomplishing this much, before proceeding to insert the plug. If the decay is in the approximal edges of the teeth, first employ the file, until the required opening is made, to enable you to proceed with facility. If it is in the grinding surface of the molars or bicuspidati, the orifice is often very small, while the disease has made great ravages in the softer substance of the tooth. In this case, proceed to enlarge the opening by the use of drills or properly pointed instruments, when we shall be able to reach the interior without difficulty. Though in this location we often find the first steps attended with some perplexity, when the decay, commencing in the centre, following the several depressions towards the outer sides of the tooth. Though the covering of these several branches is still firm and hard, they must be cut away equal to the decay in the dentine, leaving smooth and uniform edges. The preparation of such a cavity, often consumes more time than plugging; but in treating caries of the teeth, the operator should never value *time*, but use it with a becoming spirit of liberality, and the importance of the organ he claims the ability to save from the ravages of a disease that there is no temporizing with; but make the treatment both curative and preventive.

Many cavities may require but a short time, while others would consume hours of continued hard labor. Whatever price may be stipulated for so important an operation, let the operator feel determined on saving the diseased organ, and if he finds he has offered to plug a cavity for less than it is worth, let him never try to save himself at the expense of the tooth; for if half done, it had better never have been done at all, thereby saving loss to himself, expense to his patient, and last, though by no means least, dishonor to himself and the profession.

The materials capable of resisting the external agencies that act on the tooth, and suitable for plugging, we will take for granted to be only gold and tin, and that it is empirical and injurious to substitute any other. While gold is more to be dedepended upon at all times, tin may often be employed with entire confidence. The oldest plugs I have ever seen were of tin, and those had been in the mouth for forty-one years, and still perfect. Several of the molars of this gentlemen had four or five plugs in them, which had been inserted at different periods within the last half century, and of this material only, all in a sound and healthy condition, having lost but one tooth for forty-two years, and that from alveolar abscess. I regret that in the lapse of time, he has lost the name of the first operator. Albany, N. Y., has the honor of having been his field of labor. I have seen many others who have had tin stopping in their teeth from ten to twenty years, in perfect preservation.

The foil is put into different forms, for use, by operators. Some divide the sheet into several strips, and twist each; others fold them together, till it is from the fourth to a sixteenth of an inch in width; others roll it into balls; but of all forms into which I have ever prepared it, the best is by putting together six or eight sheets, and cutting into strips of the desired width. If the foil is well connected, the cut edges will adhere firmly; and if they do not, it is not fit for use.

The cavity should be well cleaned and washed out, by a lock of cotton steeped in water, and thoroughly dried with other locks of cotton, and shielded from the saliva, so as to keep both foil and tooth dry while packing the plug. With a small pointed plugger, so bent as to reach the bottom of the cavity easily, one end of the strip of foil is carried to the bottom and against the sides of the cavity, then a fold of the foil carried down by its side, and so on, fold after fold, each carried to the bottom of the cavity, and pressed firmly against the others, till the cavity is well filled, leaving each fold of the foil projecting say the eighth of an inch, according to the depth and size of the cavity. We are now prepared for consolidating the plug. For this purpose, a strong plugger, with a small needle-like point, is decidedly the best, curved or straight as the position of the cavity may be. First, make moderate pressure around the entire edge of the plug, so as to carry the projecting portions within the circle of the cavity, advancing nearer the centre every turn. After the mass is partially consolidated, the instrument, if possible, should be forced into the centre of the plug to the bottom, forcing the foil in every direction against the sides of the cavity. The cavity thus made is then filled like the first, when the instrument should be applied with much force to every part of the plug; and if at any point it can be forced into the mass, more foil must be used; and so on until it is quite solid: then proceed as before described, to consolidate the mass, applying as much force, if the cavity is large, as the tooth will bear. This should be made uniform over the entire surface, with a firm and steady hand, until the plug is perfectly solid, when the irregularities should be filed or scraped off, and more pressure applied with the plugger, so long as the least impression can be made, when the projecting portion should be filed or cut down even with the tooth. If projecting edges of the plug overlap upon the tooth, much care should be taken in cutting it away even with the walls of the cavity, when the little asperities on the surface should be smoothed down with finely powdered pummice stone, or soft wood, or floss silk; or what is much better, the Arkansas oil stone, when they can be obtained of the proper size and shape. The tooth should then be washed, and the surface well polished with a burnisher, leaving a uniform and polished surface. During the entire operation, the operator should occupy a position so as to steady the head of his patient, support the tooth, and protect the soft parts from injury or accident.

Many persons give their teeth but little thought, until in the excess of frantic pain, they are driven to a dentist for relief. The remedy is always successful if well applied—the removal of the offending organ. But this may be the farthest from the determination of the patient; and the dentist is required to "save the tooth," or at least to make a trial. The complicated caries of the teeth, is difficult, and usually perplexing, requiring patience, scientific acquirement and skill. The operator should not at once adopt the unprofessional plan of nerve killing; but endeavor to allay the irritation, protect and save it. The operator should remember that the teeth, and particularly the molares, derive much of their support through the dental canal, and by destroying the nerve the vitality of the tooth is destroyed or seriously impaired; and it soon may become but a dead and foreign substance in the mouth. In the front teeth more dependcnce can be placed upon support from the investing membrane; and in many cases they remain for many years after the loss of the pulp. Generally, if the pulp has been long exposed it becomes diseased, and an ichorous discharge is established. In this case, I doubt much the probability of arresting it, and restoring the nerve to health. The capillary vessels of the denuded pulp throw off also healthy fluid, while the debilitated state of the absorbents render it impossible for them to take it up, and send it back into the circulation. In either case stopping up the outlet by plugging the cavity in the tooth would be followed with pain as soon as the fluid had collected sufficiently to press upon the nerve; inflammation of the soft parts would soon follow with alveolar abscess. If it is simply a debilitated state of the absorbents, or enlarged capillaries, the application of galls, tannin, or a weak solution of nitrate of silver, may soon bring about a healthy action; and the tooth may be saved with its vitality by applying a gold cap over the pulp so as to prevent pressure or irritation.

But if suppuration be already established a different plan of treatment must be pursued; and it is but just to say, that they can be but temporary. Let the operator still keep in mind the importance of saving the pulp; but to accomplish this, some outlet must be left for the escape of matter. This is accomplished by capping and plugging as before mentioned, and driling through the crown of the tooth just under the margin of the gum; or packing the foil with a wire inserted through the centre of the pulp; and when the operation is completed to withdraw the wire, leaving a canal through the plug. Either plan may do well for a time; but decay must be going on underneath the plug; the disease of the pulp be increasing; and the loss of the organ is inevitable sooner or later.

Killing the nerve may do well in some cases, but the irritation caused by this unnatural treatment, is likely to extend to the investing membrane, and severe pain, inflammation and abscess soon follow. Dr. Maynard, of the District of Columbia, advises cleaning out the canal and plugging to the very apex of the root. To perform this operation properly requires a dextrous hand, and in many cases could not well be accomplished. I cannot speak of this treatment from experience, but from the pathology of the disease, and anatomy of the parts involved, it seems quite rational. There would be no accumulation of fluid or pus, and unless active inflammation supervened, would probably be absorbed as fast as secreted; or at least so long as the membrane of the socket and root remain healthy.

The plan of treatment to be pursued then, in complicated caries of the teeth, is, first, to use every effort to save the pulp in a healthy condition, protected from the plug by a cap of the same material with the plug. If not successful in this, urge the extraction of the tooth. If this fails also, adopt one of the other modes—either leave an opening for the exit of the secretions from within the canal, or destroy the nerve and plug according to the peculiarity of the case or convenience of the operator; and finally the forceps will complete the treatment.

But if there is already irritation or disease in the investing membrane or gum, particularly in the molar teeth, we are but trifling with the confidence of the patient, and abusing the good name of the profession by tampering with it.

Clarksville, Tenn., Sep. 3d 1849.

